# Does Industry Sponsorship Undermine the Integrity of Nutrition Research?

**DOI:** 10.1371/journal.pmed.0040006

**Published:** 2007-01-09

**Authors:** Martijn B Katan

## Abstract

Drug companies have tried to influence the scientific record so as to make their products look healthier. Are food companies doing the same?

The commercial success of foods depends more and more on what science says about the effects of these foods on health. Drug companies have tried to influence the scientific record so as to make their products look healthier [1,2]; are food companies doing the same? In a study published in *PLoS Medicine*, Lesser et al. [3] investigated this by analyzing 206 publications on the health effects of milk, soft drinks, and fruit juices. Twenty-four of these studies had been funded solely by the industry whose product was investigated, while 52 of the papers declared that they had had no industrial support. The other papers had mixed support or did not declare sponsorship.

The odds that a paper would report a favorable outcome were four to eight times higher when the study was funded by the manufacturer of the beverages in question than when the study was not funded by industry. Out of the 35 interventional studies, which included human trials, industry was the sole sponsor of 16, and none of these 16 reported an unfavorable outcome. In contrast, seven of the 19 interventional studies with mixed or no industry funding found an unfavorable effect. Thus, papers sponsored by industry were more likely to report favorable outcomes for that industry's beverages than papers with other sources of funding.

The study by Lesser et al. was carefully done, the number of articles was sufficiently large, the analyses were straightforward, and they agree with the outcomes of earlier, smaller studies. However, an association between funding and outcome does not by itself prove bias. First, five of the papers dealt with outbreaks of food poisoning and none of these studies was funded by industry, which strengthened the correlation of unfavorable outcomes with the absence of industry funding. But industry was, of course, never asked to fund these studies and therefore bias was not an issue. Second, when producers plan to fund a nutrition study, they will naturally select a product with a potentially favorable nutritional profile.

Drug companies have tried to influence the scientific record so as to make their products look healthier; are food companies doing the same?

However, such selection is the start of a slippery slope. When an industry is the major sponsor of research on its own product, unfavorable effects of that product are less likely to be investigated. The next step down the slope is adjustment of designs. The dosage of the product and the nature of control treatments may be adjusted so as to increase the chance that the study will demonstrate benefits of the product or that adverse effects will not reach statistical significance. Also, unfavorable data may be deemed less relevant and may be left out of the abstract and the press release, or out of the paper itself. Finally, the whole publication may be cancelled or seriously delayed when the outcome is disappointing to the sponsor. Innocuous-sounding clauses in the contract may give the company such a veto right, and investigators may not fully realize the consequences of what they are signing. Some contract research organizations grant the sponsor that veto right up front. Even if researchers can legally publish the data, they may be reluctant to antagonize a major sponsor.

There are indications that all these things happen [4,5], but there are few hard quantitative data to prove it. As Marion Nestle said in her landmark book [5]: “I could not find anyone who would speak to me ‘on the record’ for this book. When I told friends in government, food companies, and academia that I was writing a book about how the food industry affects nutrition and health, they offered to tell me anything I wanted to know, but not for attribution.” We obviously need more studies of the relations between industry and nutrition research, and they may need to go beyond the data made public in scientific journals. Meanwhile, what should we do? My personal experience makes me reluctant to support a blanket condemnation of industry-supported research, because collaboration with industry has allowed me to discover things that I could not have found otherwise. We discovered the effects of trans fatty acids on heart disease risk [6] thanks to the expertise of Unilever, and the cholesterol-raising factor in unfiltered coffee [7] thanks to Nestlé.

But researchers dealing with industry may be subjected to pressure, and they need help to resist such pressure. Most universities now have a code of conduct on relations with industry and conflicts of interest, but when the negotiations come down to the wire, and money and jobs are at stake, then a code of conduct may not be enough to keep a researcher on the straight and narrow. The Royal Netherlands Academy of Sciences has put forth an innovative proposal on how to supervise relations between researchers and their sponsors [8]. For now, the Lesser et al. study raises serious concerns that some food industries may distort the scientific record on diet and health. Such concerns affect nutrition science as a whole, if only because they threaten public confidence in nutrition research, and once that confidence is gone nutrition research becomes irrelevant.
